# Prenatal and Postnatal Identification of Phelan–McDermid Syndrome in a Tertiary Referral Center: A 15-Case Series and Literature Review

**DOI:** 10.3390/jcm15187130

**Published:** 2026-09-14

**Authors:** Huili Xue, Yifang Dai, Xianglan Ye, Lin Zhang, Qun Guo, Na Lin, Hailong Huang, Liangpu Xu

**Affiliations:** 1 Medical Genetic Diagnosis and Therapy Center, Fujian Key Laboratory for Prenatal Diagnosis and Birth Defect, Fujian Maternity and Child Health Hospital, College of Clinical Medicine for Obstetrics & Gynecology and Pediatrics, Fujian Medical University, No. 18 Daoshan Road, Gulou District, Fuzhou 350001, China; huilixue2026@fjsfy.com (H.X.); daiyifang379@fjmu.edu.cn (Y.D.); 728762486@fjmu.edu.cn (Q.G.); linna1088@fmu.edu.cn (N.L.); 2 College of Obstetrics, Gynecology and Pediatrics, Fujian Medical University, Wushan Campus, No. 88 Jiaotong Road, Taijiang District, Fuzhou 350001, China; 15750846366@fjmu.edu.cn (X.Y.); xinfang@fjmu.edu.cn (L.Z.)

**Keywords:** 22q13 deletion syndrome, *SHANK3*, prenatal diagnosis, copy number variation, genotype–phenotype correlation

## Abstract

**Objectives**: To characterize the clinical and genetic features of Phelan–McDermid syndrome (PMS) in a Chinese prenatal and postnatal cohort, explore genotype–phenotype correlations, and provide evidence to support prenatal genetic counseling. **Methods**: G-banded karyotyping, single-nucleotide polymorphism arrays (SNP arrays), copy number variation sequencing, and trio whole-exome sequencing were used for genetic diagnosis. Fifteen patients with PMS (11 prenatal and 4 postnatal) were retrospectively enrolled. Genomic visualization, protein structural prediction, and phenotypic heatmap analyses were conducted to analyze genotype–phenotype associations. **Results**: The prenatal and postnatal detection rates of PMS were 0.061% and 0.49%, respectively. Only 13.3% of cases were detected by karyotyping, whereas 14 cases were confirmed by SNP array analysis. All 22q13 deletions (69.4 kb–8.5 Mb) involved *SHANK3*, and all copy number variants were de novo. A novel *SHANK3* frameshift variant, c.3513_3514delCC, was identified and predicted to result in protein truncation leading to intellectual disability. Prenatal cases mainly presented non-specific ultrasound anomalies (63.6%, 7/11), with fetal growth restriction, renal malformations, and cardiovascular defects being the most frequent findings. A notable 36.4% (4/11) of prenatal cases were complicated by missed abortion, whereas postnatal patients showed predominant neurodevelopmental impairments. In our cohort, larger deletions were observed in cases presenting with more extensive multisystem involvement, a pattern that mirrors findings from recent large-cohort studies. central nervous system abnormalities were predominantly linked to *SHANK3* haploinsufficiency, while multi-system malformations seemed to correlate with the cumulative loss of multiple genes across the 22q13 region, in keeping with current hypotheses on contiguous gene effects. **Conclusions**: PMS presents obvious prenatal–postnatal phenotypic heterogeneity. *SHANK3* is the core pathogenic gene, and deletion may influence the extent of multisystem involvement. Combined genetic testing can improve the accuracy of prenatal diagnosis and counseling for PMS.

## 1. Introduction

Phelan–McDermid syndrome (PMS), also known as 22q13 deletion syndrome (Online Mendelian Inheritance in Man (OMIM) #606232) [1], is a rare multisystem genetic disorder characterized by substantial clinical and genetic heterogeneity [2]. The core clinical manifestations of PMS include developmental delay (DD), intellectual disability (ID), absent or delayed speech, autism spectrum disorder (ASD), neonatal hypotonia, behavioral abnormalities, and characteristic dysmorphic facial features [3,4].

PMS is primarily caused by microdeletions involving chromosome 22q13 or pathogenic variants in *SHANK3* (OMIM 606230). *SHANK3*, a ~58 kb gene located at chromosome 22q13.33 that comprises 25 exons encoding a 1747-amino acid scaffold protein [5], is highly enriched in the postsynaptic density of excitatory synapses [6], where it anchors membrane-bound receptors to the actin cytoskeleton and plays a pivotal role in synaptic function and dendritic development [7]. Haploinsufficiency of *SHANK3* is the primary molecular mechanism underlying PMS [8] and accounts for the broad spectrum of neurological phenotypes observed in patients [9,10].

Beyond the core role of *SHANK3* haploinsufficiency in driving neurodevelopmental phenotypes, recent large-scale cohort studies have established that deletion size is a critical determinant of multisystem manifestations in PMS, with larger deletions consistently associated with renal malformations, congenital heart defects, and more severe global functional impairment [11,12,13]. Moreover, several candidate genes located proximal to *SHANK3* have been proposed to modulate the core clinical phenotype, and the cumulative haploinsufficiency of multiple dosage-sensitive genes along 22q13 is thought to underlie the multisystem involvement [14,15]. However, systematic analyses of genotype–phenotype correlations specifically in prenatally diagnosed PMS cohorts—particularly in the Chinese population—remain scarce.

While postnatal features of PMS have been well documented in numerous studies, prenatal manifestations of PMS are often nonspecific, making early clinical recognition challenging. To date, only a limited number of prenatal PMS cases have been reported, with most studies relying on small case series or individual case reports. The largest prenatal cohort to date, described by Hao et al., included 21 cases and highlighted the predominance of non-specific ultrasound anomalies such as fetal growth restriction, renal malformations, and cardiac defects [16]. Similarly, Nevado et al., in a multicenter cohort of 210 individuals, observed that prenatal cases frequently presented with non-specific structural malformations, and approximately 85% of prenatally diagnosed pregnancies were terminated after genetic counseling [11]. These observations underscore the diagnostic challenges posed by the lack of distinctive prenatal features and emphasize the need for comprehensive genetic testing in fetuses with unexplained structural anomalies. However, whether the extent of prenatal ultrasound anomalies correlates with specific deletion sizes or gene content within the 22q13 region remains largely unexplored.

To address this gap and further delineate the clinical and genetic spectrum of PMS, we retrospectively analyzed 11 prenatally diagnosed and 4 postnatally diagnosed Chinese patients with PMS identified at our tertiary referral medical center. Comprehensive genetic testing, including karyotyping, single-nucleotide polymorphism array (SNP array) analysis, copy number variation (CNV) sequencing, and whole-exome sequencing (WES), was performed.

## 2. Materials and Methods

### 2.1. Patients and Samples

Between June 2016 and March 2025, a total of 18,160 prenatal samples and 820 postnatal samples underwent genetic testing at the Fujian Key Laboratory for Prenatal Diagnosis and Birth Defects, Fujian Maternity and Child Health Hospital. Baseline clinical characteristics, prenatal diagnostic indications, prenatal ultrasound findings, pregnancy outcomes, clinical manifestations, and genetic test results were retrospectively collected and analyzed for the 15 patients diagnosed with PMS.

Biological specimens included amniotic fluid, chorionic villous tissue from terminated pregnancies, and peripheral blood samples. This study was approved by the Institutional Ethics Committee of Fujian Maternity and Child Health Hospital, Fujian Province, China. All pregnant participants or the guardians of affected children received pretest genetic counseling and completed standardized questionnaires covering demographic information, age at symptom onset, clinical manifestations, medical history, auxiliary examination findings, and family history.

### 2.2. G-Banded Karyotyping

G-banded karyotyping was conducted on cultured amniotic fluid cells, aborted villous tissues and peripheral blood lymphocytes following the standard laboratory protocols established in our center, with a resolution ranging from 320 to 500 bands. A minimum of 20 metaphase spreads were counted, and five karyograms were meticulously analyzed. All chromosomal abnormalities were designated in compliance with the International System for Human Cytogenomic Nomenclature 2020.

### 2.3. SNP Array

SNP array was implemented using the Affymetrix CytoScan 750K platform (Affymetrix Inc., Santa Clara, CA, USA). Raw data were processed via the Affymetrix Chromosome Analysis Suite software (version 3.1.0.15), consistent with our previously published protocol [17]. The reporting cutoff was defined as copy number gains or losses ≥ 400 kb and regions of loss of heterozygosity ≥ 10 Mb. All identified CNVs were cross-referenced against our in-house database and multiple public genomic repositories, including the Database of Genomic Variants (DGV) (http://dgv.tcag.ca/dgv/app/home (accessed on 31 March 2025)), DECIPHER (https://www.deciphergenomics.org/ (accessed on 31 March 2025)), the International Standards for Cytogenomic Arrays Consortium (ISCA) (https://www.iscaconsortium.org/ (accessed on 31 March 2025)), ClinVar (https://www.ncbi.nlm.nih.gov/clinvar/ (accessed on 31 March 2025)), the ClinGen Dosage Sensitivity Database (https://dosage.clinicalgenome.org (accessed on 31 March 2025)), and OMIM. Each CNV was categorized as pathogenic, likely pathogenic (LP), variant of uncertain significance (VUS), likely benign, or benign in accordance with the 2019 American College of Medical Genetics and Genomics (ACMG) variant classification guidelines [18].

### 2.4. CNV Sequencing

CNV sequencing (CNV-seq) was initially conducted for Postnatal Case 4 using peripheral blood collected at 2.5 years of age. Briefly, genomic DNA was fragmented, followed by end repair, adapter ligation, and PCR amplification to construct sequencing libraries. Single-end sequencing was carried out on the NextSeq CN500 platform (Annoroad Gene Technology Co., Ltd., Beijing, China). After quality control filtering, each sample generated more than 8 million clean reads with an average sequencing depth of approximately 1×. All sequencing tags were mapped to the human reference genome, and standardized Z-score analysis was adopted to identify chromosomal copy number variations. Unique mapped reads were aligned to the hg19 human reference genome via the Burrows–Wheeler algorithm and assigned to consecutive 20 kb bins across all chromosomes [19]. The reporting threshold for CNV calling was set at copy number gains or losses of ≥100 kb. Detected CNVs were cross-referenced against multiple public databases, including the DGV, 1000 Genomes Project, DECIPHER, ISCA, PubMed, the UCSC Genome Browser, ClinVar, the ClinGen Dosage Sensitivity Database, and OMIM. The pathogenicity of each CNV was classified in accordance with the ACMG variant interpretation guidelines [18].

### 2.5. WES and Bioinformatic Analysis

Trio-WES was performed for Postnatal Cases 3 and 4 to exclude causative monogenic disorders. Genomic DNA libraries were constructed with the Agilent SureSelectXT Human All Exon V6 capture kit (Agilent Technologies, Santa Clara, CA, USA). Paired-end sequencing was performed on the Illumina NovaSeq 6000 platform (Illumina, San Diego, CA, USA) following the manufacturer’s standard protocols.

In silico pathogenicity prediction was implemented using three tools: MutationTaster (https://www.mutationtaster.org (accessed on 31 March 2025)), PolyPhen-2 (http://genetics.bwh.harvard.edu/pph2 (accessed on 31 March 2025)), and CADD (https://cadd.gs.washington.edu (accessed on 31 March 2025)). All single-nucleotide variants (SNVs) and small insertions/deletions (indels) were annotated against multiple public databases and literature resources, including PubMed (https://pubmed.ncbi.nlm.nih.gov/ (accessed on 31 March 2025)), the Genome Aggregation Database (gnomAD, http://gnomad.broadinstitute.org (accessed on 31 March 2025)), the 1000 Genomes Project (http://www.1000genomes.org/ (accessed on 31 March 2025)), the Exome Aggregation Consortium (ExAC, https://gnomad.broadinstitute.org/ (accessed on 31 March 2025)), the Human Gene Mutation Database (HGMD, http://www.hgmd.cf.ac.uk/ac/index.php (accessed on 31 March 2025)), ClinVar, and OMIM. Variant pathogenicity was interpreted in accordance with the ACMG variant classification guidelines [20]. All candidate pathogenic variants were validated via Sanger sequencing. All variants were reviewed by a multidisciplinary team including clinical geneticists, prenatal diagnostic specialists, obstetricians, pediatricians, neonatologists, bioinformaticians, and radiologists. CNVs identified from the WES data were analyzed using two commercial software packages: WES Master 2.0 and NxClinical 6.1.

### 2.6. Visualization of CNV Profiles Across Multiple Samples

SNP array data from all 15 enrolled samples were processed, and CNVs located outside chromosome 22 were excluded from subsequent analyses. CNV metadata, including chromosomal location, start and end genomic coordinates, and variant types, were compiled and visualized using the Gviz R package (version 1.38.0) [21]. Ideogram and genomic axis tracks were constructed using the UCSC hg19 human reference genome. Protein-coding genes were retrieved using the biomaRt R package (Ensembl release 105) [22] and displayed as light-blue BiomartGeneRegionTrack objects. For each patient, an annotation track was generated, with deletions shown in red and duplications in blue. The plotting range was automatically extended by 10% of the CNV length or by a minimum of 50 kb beyond the outermost breakpoints. The genomic coordinates of *SHANK3* were extracted from the annotation track, and two vertical dashed lines were added using the grid framework to delineate the gene boundaries and determine whether the CNV encompassed *SHANK3*.

### 2.7. Comparative Tertiary Structure Prediction of SHANK3

The human wild-type SHANK3 sequence (1748 amino acids, UniProt ID Q9BYB0, isoform 1) was retrieved from UniProt. The c.3513_3514delCC frameshift variant (p.L1172Hfs*198) was constructed based on the sequencing results. This variant introduces a premature termination codon at amino acid 1370, resulting in a truncated protein of 1370 amino acids. Wild-type and mutant sequences were submitted to the SWISS-MODEL server (https://swissmodel.expasy.org/ (accessed on 31 March 2025)) [23] for three-dimensional structural homology modeling. Multiple templates were used to model the full-length structure of wild-type SHANK3, whereas the mutant protein was modeled independently. Ligands and water molecules were excluded from all structural models.

The resulting PDB files were imported into Swiss-PdbViewer (version 4.10) for structural alignment and comparison [24]. Structural superposition was performed using the backbone atoms of residues 1–1171 (the region upstream of the frameshift site), corresponding to the region upstream of the frameshift site, to evaluate structural alterations induced by the variant. Secondary structural features were annotated automatically by the software.

### 2.8. Heatmap Analysis of Phenotype Proportions Across Candidate Genes and Clinical Organ Systems

All 15 enrolled patients, including both prenatal and postnatal cases, were included in the analysis. Clinical phenotypes were classified according to organ system involvement, including the central nervous system (CNS), musculoskeletal system (MSK), cardiovascular system (CVS), genitourinary system (GUS), gastrointestinal system (GIS), endocrine and metabolic system (EMS), sensory organs (SOs), developmental facial features (DF), behavioral and growth abnormalities (BG), and other clinical manifestations.

CNVs identified by chromosomal microarray analysis (CMA) were annotated to identify all protein-coding genes within each deleted region. For each gene–organ system pair, phenotypic prevalence was calculated as the proportion of patients with deletions involving the corresponding gene who exhibited the relevant clinical manifestation. Heatmaps were generated using the Python library Seaborn (v0.13.2) and matplotlib (v3.10.9), with white and red indicating low and phenotypic proportions, respectively.

### 2.9. Follow-Up

All prenatal patients underwent telephone follow-up to determine obstetric outcomes. Postnatal patients were followed regularly by a pediatric neurologist, and growth and neurodevelopmental data were obtained through telephone interviews and reviews of medical records. Follow-up assessments focused on physical growth and neurodevelopmental status, with the final follow-up date set as 6 December 2025.

## 3. Results

### 3.1. General Clinical Characteristics

A total of 18,160 prenatal and 820 postnatal samples underwent genetic testing at our center. Eleven prenatal fetuses and four postnatal patients were molecularly diagnosed with PMS, corresponding to detection rates of 0.061% and 0.49%, respectively.

Among the 11 pregnant cases, maternal age ranged from 20 to 44 years, and gestational age at diagnosis ranged from 11 to 25w6d. All parents were non-consanguineous and had unremarkable medical histories. None of the mothers reported teratogenic exposure or infectious diseases during pregnancy. Baseline demographic and clinical data of all patients with PMS are summarized in Table 1, Table 2 and Table 3.

### 3.2. Phenotypic Characteristics

Among the 11 prenatal PMS cases, four resulted in missed abortion. The remaining seven pregnancies underwent prenatal diagnosis because of advanced maternal age or ultrasonic abnormalities. All seven fetuses exhibited abnormal prenatal ultrasound findings, including fetal growth restriction and multiple craniofacial developmental anomalies, such as decreased biparietal diameter (BPD), reduced head circumference (HC), mildly diminished cerebellar transverse diameter, and bilateral ventriculomegaly. Urinary tract abnormalities included bilateral polycystic renal dysplasia, absent bladder, left polycystic renal dysplasia, left hydronephrosis, mildly increased bilateral renal parenchymal echoes, and bilateral accessory renal arteries. Skeletal abnormalities included shortened femoral length and abnormal spinal development. Cardiac abnormalities included ventricular septal defect, transiently reduced cardiac systolic function, persistence left superior vena cava (PLSVC), left ventricular echogenic focus, and mild tricuspid regurgitation. One fetus also presented with gallbladder agenesis (Table 2).

Among the four postnatal patients, two presented with global developmental delay (GDD) accompanied by severe speech impairment. The remaining two were diagnosed with ID, one of whom also exhibited autistic features, myasthenia, and muscular dystrophy (Table 3).

### 3.3. Cytogenetic Results

Successful G-banded karyotyping was performed in all 15 patients. Chromosomal abnormalities involving 22q13.2 deletions were identified in only two cases, corresponding to a detection rate of 13.3% (2/15). No derivative chromosome 22 resulting from parental balanced translocations was identified. Detailed cytogenetic findings are summarized in Table 2 and Table 3.

### 3.4. SNP Array and CNV-Seq Results

SNP array analysis confirmed PMS in 14 patients. Twelve patients (80.0%, 12/15) harbored 22q13.1q13.3 deletions involving SHANK3, whereas Prenatal Case 4 exhibited high-level mosaicism (70%). Two patients (Postnatal Cases 1 and 4) carried intragenic SHANK3 deletions spanning exons 12–25. Prenatal Case 2 carried a derivative chromosome 22 with 22q13.2q13.31 duplication and a concurrent 22q13.31q13.33 deletion. Deletion sizes of 22q13.1q13.3 ranged from 69 kb to 8.5 Mb.

In addition, SNP array identified a pathogenic mosaic duplication of 15q13.3q24.3 (70% mosaicism) in Prenatal Case 4. Variants of uncertain significance were detected in Prenatal Cases 2 and 9. All pathogenic CNVs associated with PMS occurred de novo.

**Figure 1:**
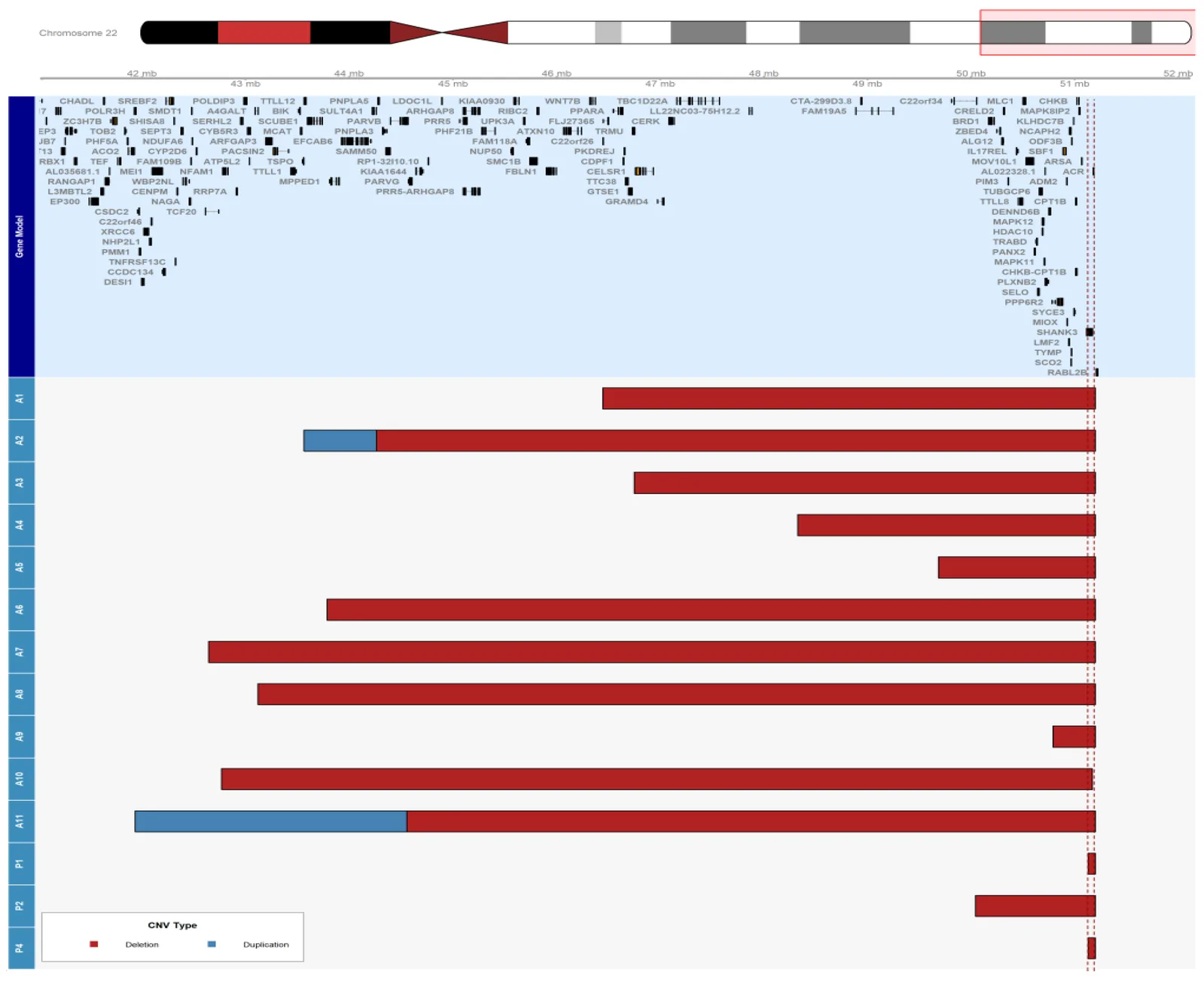
Copy number variations on chromosome 22 across 15 cases. The figure shows the deleted and duplicated regions on chromosome 22 for 15 cases. The top track displays the chromosome ideogram and a genomic ruler; the middle track shows protein-coding genes in the region (light blue); the bottom track shows the deletion and duplication regions for each sample. All results are based on hg19. Each sample (A1–A11 corresponds to cases 1–11, P1–P4 corresponds to cases 12–15) has its own row: red boxes represent deletions, blue boxes represent duplications. The two dashed red vertical lines indicate the start and end of the *SHANK3* gene (chr22:51,128,325–51,197,766). Most samples carry a large deletion extending to the telomere; cases A2 and A11 contain an additional proximal duplication.

For Postnatal Case 4, CNV-seq of peripheral blood initially revealed a 0.44 Mb duplication at chr15:g.32,020,000_32,460,000 [seq(hg19) dup(15)(q13.3q13.3)]. Subsequent SNP array analysis identified a 69.4 kb heterozygous deletion at arr[hg19] 22q13.33 (51,128,325_51,197,766) ×1. Detailed genetic findings are summarized in Tables S2 and S3, and the genomic profiles of all 22q CNVs are shown in Figure 1.

### 3.5. WES Findings

Trio-WES was conducted for two postnatal patients after conventional karyotyping, and CMA and CNV-seq failed to identify pathogenic large-scale genomic rearrangements. In Postnatal Case 3, which presented with moderate ID, WES identified a de novo heterozygous frameshift variant, *SHANK3* c.3513_3514del (p.L1172Hfs*198), in exon 21 (transcript NM_001372044.1). Consistent with the autosomal dominant inheritance mode of PMS, this likely pathogenic variant introduced a premature stop codon at amino acid 1370, resulting in a truncated SHANK3. The wild-type SHANK3 structure exhibits regularly arranged α-helices and β-sheets (Figure S1A), whereas in the mutant protein, structural modeling predicted marked loss of the C-terminal α-helical and β-sheet domains (marked by the red arrow in Figure S1B). These structural changes may severely impair multiple functional domains, including the homoserine-binding motif, cortactin-binding region, and sterile alpha motif domain.

For Postnatal Case 4, trio-WES was performed to further investigate the variant of uncertain significance duplication at seq[hg19]dup(15)(q13.3q13.3) (chr15:g.32,020,000_32,460,000) detected by CNV-seq. WES identified a 0.11 Mb pathogenic intragenic deletion at 22q13.33 (chr22: 51,123,013–51,237,556) involving exons 11–25 and the 3′ terminal region of *SHANK3*, as well as adjacent *ACR* and *RABL2B*. This deletion was subsequently confirmed by SNP array analysis. WES also detected a maternally inherited hemizygous missense variant of uncertain significance in *VMA21* (c.214C>T (p.R72C); minor allele frequency 0.00184316). This variant predisposes the patient to myopathy accompanied by excessive autophagy.

### 3.6. Genotype–Phenotype Correlation Analysis of 22q13 Deletion Syndrome

To explore the contribution of individual genes within the 22q13 critical region to the diverse clinical manifestations of PMS, we performed gene-centered genotype–phenotypic association analysis. For each protein-coding gene within the deleted interval, we calculated the number of deletion carriers exhibiting abnormalities across 10 major organ systems (Tables S1 and S2). The CNS was the most frequently affected, followed by BG abnormalities (predominantly fetal growth restriction (FGR), together with reduced BPD, HC, and femoral length), other clinical outcomes (including oligohydramnios and missed abortion), the GUS, and the CVS (Table S1).

Among the 15 patients, six (40.0%) exhibited CNS-related phenotypes, including GDD, ID, language impairment, cerebral white matter myelination defects, ASD, and ventriculomegaly. Other commonly affected systems in *SHANK3*-deleted patients included the miscellaneous category (5/15, 33.3%, mainly FGR and missed abortion), GUS (4/15, 26.7%, including polycystic kidney disease and hydronephrosis), CVS (4/15, 26.7%, including ventricular septal defect, PLSVC, and cardiac echogenic foci), and BG anomalies (4/15, 26.7%, primarily FGR). Musculoskeletal system abnormalities and dysmorphic facial features were each observed in two patients. No endocrine and metabolic system or sensory organ (visual and auditory) abnormalities were identified (Table S2).

At the system level, certain patterns of genotype-phenotype association emerged across the 22q13 region. *SHANK3* deletion (*n* = 15) was associated with the highest frequency of CNS involvement (6/15, 40.0%), whereas CNS manifestations were less frequent among carriers of deletions involving *ACR* (5/13, 38.5%) or other recurrently deleted genes (3/12, 25.0%; *ADM2*, *ARSA*, *CHKB*, *CPT1B*, *KLHDC7B*, *LMF2*, *MAPK8IP2*, *MIOX*, *NCAPH2*, *PPP6R2*, *SBF1*, *SCO2*, *SYCE3*, and *TYMP*). Musculoskeletal system abnormalities were relatively rare and predominantly observed in patients harboring larger genomic deletions. CVS and GUS manifestations were common, with phenotypic positivity rates ranging from 30% to 50% for most codeleted genes, including *ACR*, *ADM2*, *ARSA*, *CHKB*, *CPT1B*, *KLHDC7B*, *LMF2*, *MIOX*, *NCAPH2*, *PPP6R2*, *SBF1*, *SCO2*, *SYCE3*, and *TYMP* (Figure S2). Specifically, *CELSR1*, *CERK*, *GRAMD4*, *TBC1D22A*, and *TRMU* exhibited phenotypic rates of 50.0% (4/8), whereas *TAFA5* exhibited positivity rates of 44.4% (4/9) for GUS anomalies and 33.3% for CVS involvement. Gastrointestinal abnormalities were uncommon, with the most frequently deleted genes associated with a phenotypic prevalence of 20–25%; only *TCF20* deletion (*n* = 1) was concurrently linked to gastrointestinal system and GUS abnormalities (Table S2 and Figure S2). Developmental facial features were exclusively identified in postnatal patients, occurring in 13.3% of *SHANK3* deletion carriers (2/15) and approximately 15% of carriers of other recurrently deleted genes. BG abnormalities (notably FGR) were common across multiple genes, with prevalence ranging from 26.7% to 33.3%. Phenotypes classified into the miscellaneous group (oligohydramnios and missed abortion) were observed in 33.3% of *SHANK3* deletion carriers and reached 41.7–55.6% for other frequently deleted genes.

Collectively, these observations align with the prevailing view that *SHANK3* is centrally involved in the neurodevelopmental manifestations of PMS. In contrast, CVS, GUS, BG, and other multisystem abnormalities appeared to reflect the combined effects of larger deletions involving multiple dose-sensitive genes rather than loss of a single causal gene.

### 3.7. Outcome and Follow-Up

Among the 11 prenatal cases with 22q13.3 deletions, four resulted in missed abortion, and the remaining seven pregnancies were electively terminated. Four postnatal patients with PMS underwent long-term follow-up from ages 2.5 to 10 years. All patients exhibited neurodevelopmental disorders of varying severity, including GDD, DD, moderate-to-severe ID, gross motor delay, ASD, and muscular dystrophy. Detailed prenatal and postnatal follow-up data are presented in Table 2 and Table 3.

## 4. Discussion

To our knowledge, this study represents the largest reported cohort focusing specifically on prenatal PMS. We reviewed the clinical characteristics and diagnostic findings of prenatally diagnosed PMS cases over the past 24 years (Table S3). In our cohort, prenatal PMS was predominantly characterized by non-specific ultrasound abnormalities (UAs), including FGR, renal malformations (polycystic dysplastic kidney and hydronephrosis), cardiovascular anomalies, gastrointestinal abnormalities, and CNS defects. Notably, the frequencies of non-specific ultrasound anomalies (63.6%) and missed abortion (36.4%) in our prenatal PMS cohort were substantially higher than those reported in the general fetal population (2–3% [25] and 6.8% [26], respectively), underscoring the broad spectrum of prenatal presentations. These findings are consistent with those reported by Hao et al. [16] and Nevado et al. [11].

In contrast, all postnatally diagnosed patients exhibited the characteristic neurodevelopmental phenotype of PMS, including GDD, ID, language delay, and ASD, accompanied by dysmorphic facial features and hypotonia. Three of these patients carried relatively small 22q13.3 deletions, whereas one harbored a frameshift variant in *SHANK3*. The striking difference between the prenatal and postnatal phenotypic spectra primarily reflects the fact that neurodevelopmental manifestations—the hallmark features of PMS—are not amenable to prenatal detection by ultrasound, whereas structural anomalies are the main observable findings in utero. This observation underscores the inherent diagnostic limitation of prenatal imaging and reinforces the critical role of molecular testing in fetuses with unexplained structural anomalies, particularly when multiple systems are involved.

Genomic profiling revealed heterozygous 22q13 deletions in 14 of the 15 cases, with deletion sizes ranging from 69.4 kb to 8.5 Mb and encompassing the terminal 22q13.33 region containing *SHANK3*. This distribution closely resembles that reported in international cohorts, in which deletions ranged from approximately 10 kb to 9 Mb and were predominantly clustered within the subtelomeric 22q13.3 region [15]. The smallest overlapping deleted region in our cohort encompassed chr22:51128325–51197766 (hg19) containing only *SHANK3* and its adjacent sequences, further supporting *SHANK3* as the principal disease-associated gene in PMS [27,28]. One patient carried a novel SHANK3 frameshift variant (c.3513_3514delCC, p.Leu1172GlyfsTer139), which resulted in truncation of the C-terminal functional domain of SHANK3 and is predicted to disrupt synaptic scaffold protein–protein interactions (Figure S1). This structural prediction provides a plausible explanation for the patient’s moderate ID and is consistent with previous reports demonstrating that C-terminal truncating variants of SHANK3 produce loss-of-function effects associated with severe neurodevelopmental phenotypes [7]. Although *SHANK3*-sparing deletions have previously been associated with PMS-like phenotypes, such as ID and speech delay [29,30], no such cases were identified in our cohort, likely reflecting their rarity and the limited sample size. Two prenatal cases also harbored additional copy number abnormalities, a 699 kb duplication spanning 22q13.2–q13.31 and a 2.6 Mb tetrasomy in the same chromosomal interval [28,31]. These results suggest that this subtelomeric segment is highly susceptible to structural genomic rearrangements. Although these secondary rearrangements may contribute to adverse neurodevelopmental outcomes, the limited number of reported cases precludes firm conclusions regarding their clinical significance [32,33].

From a phenotypic perspective, CNS manifestations were the most frequent abnormalities and were strongly associated with *SHANK3* haploinsufficiency, consistent with previous studies identifying *SHANK3* as the principal neurodevelopmental gene underlying PMS [15,34]. In contrast, cardiovascular, genitourinary, and growth abnormalities appeared to correlate with larger deletions involving multiple dosage-sensitive genes. These findings are broadly in line with several recent cohort studies, which have similarly noted that larger deletion size often corresponds to a broader range of systemic involvement [35]. Gene-based phenotypic correlation analyses indicated that multisystem structural malformations were correlated with the cumulative haploinsufficiency of numerous genes across the 22q13 region instead of pathogenic variants in individual genes outside the *SHANK3* locus [15]. This gene-dosage model has received considerable support from multiple independent cohorts, although the exact contribution of individual genes outside *SHANK3* warrants further exploration. Our study further suggests that CVS and GUS abnormalities were more frequent among patients with deletions involving *CELSR1*, *CERK*, *GRAMD4*, *TRMU*, and *TAFA5*, with the prevalence of these structural abnormalities ranging from 33.3% to 50.0%. These observations are consistent with those of previous studies implicating *CELSR1* and *UPK3A* in renal and cardiovascular development, supporting their potential roles as phenotypic modifiers of cardiac and urogenital manifestations in PMS [15,36]. FGR has been reported sporadically in prenatally diagnosed PMS, suggesting that gene dosage across the 22q13 region influences fetal growth during development [34]. In our cohort, more than one-third of patients with deletions encompassing *ACR*, *ADM2*, and *ARSA* exhibited FGR, raising the possibility that these loci may be involved in pathways influencing prenatal growth. Musculoskeletal abnormalities were identified in only two patients (Prenatal Case 9 and Postnatal Case 4), both of whom harbored relatively small deletions (410 and 69.4 kb, respectively). This result suggests that isolated *SHANK3* haploinsufficiency can trigger musculoskeletal phenotypes, consistent with previous studies indicating that *SHANK3* deficiency alone contributes to neuromuscular abnormalities, including hypotonia, through impaired neuromuscular junction formation and striated muscle maturation [37]. Overall, our findings further support *SHANK3* as the principal gene underlying the neurodevelopmental manifestations of PMS and suggest that deletion size contributes substantially to multisystem involvement. Except for *SHANK3*, few single genes on 22q13 may independently trigger multisystem clinical phenotypes. Instead, cumulative haploinsufficiency arising from contiguous gene deletions is likely the primary molecular mechanism underlying these multisystem manifestations.

The observed phenotypic differences between prenatally and postnatally diagnosed PMS cases carry important clinical implications. For prenatal counseling, the broad spectrum of potential outcomes—ranging from intrauterine demise to survival with variable neurodevelopmental disabilities—should be communicated to families, consistent with the 85% termination rate reported by Hao et al. (2022) [16]. For prognostic assessment, the universal presence of neurodevelopmental disorders in all live-born patients (100%) reinforces the necessity of early neurodevelopmental surveillance and long-term follow-up planning. For postnatal management, the correlation between deletion size and multisystem involvement highlights the importance of precise genetic characterization to guide individualized monitoring and multidisciplinary care, particularly for cardiac and renal complications, as emphasized in the European PMS consortium consensus recommendations (2023) [38]. Our findings highlight the clinical value of CMA for fetuses presenting with multiple structural abnormalities or adverse pregnancy histories, as the characteristic neurodevelopmental manifestations of PMS cannot be detected by prenatal imaging alone [23]. Based on these observations, we recommend CMA as the first-line diagnostic test for fetuses with unexplained FGR or structural anomalies, particularly when multiple systems are involved, given its high detection rate (93.3%) in our cohort compared to karyotyping (13.3%). If CMA is negative but clinical suspicion persists, trio-WES or WGS may be considered as a sequential testing option. The proposed diagnostic pathway includes: (i) detailed targeted ultrasound examination; (ii) pre-test genetic counseling; (iii) CMA as the primary test; (iv) trio-WES/WGS as a secondary test if CMA is negative; (v) post-test genetic counseling on results, prognosis, and reproductive options; and (vi) multidisciplinary team involvement for comprehensive management.

Our findings expand the known clinical and genetic spectrum of PMS in both prenatal and postnatal settings and provide additional evidence supporting CMA and next-generation sequencing for fetuses presenting with UAs suggestive of PMS. Earlier molecular diagnosis may improve prenatal counseling, facilitate clinical decision-making, and enhance postnatal management and long-term follow-up.

The limitations of our study include the limited sample size, retrospective single-center cohort study design, risks of referral bias and case-detection bias, as well as the small number of postnatally diagnosed cases, and long-term follow-up data were also limited. Larger multicenter studies with standardized phenotyping and extended follow-up are needed to validate these findings and improve genetic counseling.

## Figures and Tables

**Table 1:** Summary of genetic results of 15 cases with Phelan-McDermid Syndrome.

Type of Genetic Variants	*n* (%)
Simple 22q13 deletions including *SHANK3*	11 (73.33)
Intragenic deletion in *SHANK3*	2 (13.33)
postzygotic mosaic deletion	1 (6.67)
Sequence variant in *SHANK3*	1 (6.67)

**Table 2:** Details of the 22q13.3 deletions encompassing more than *SHANK3* gene detected in prenatal 11 cases.

Prenatal Case	Gestational Age at Diagnosis(w)	Karyotype	SNP Array Results (Inheritance)	CNV Size	Number of OMIM Genes	Ultrasonic Findings/Prenatal Diagnostic Indication	Pregnancy Outcome
1	13+	46,XY	arr[hg19]22q13.31q13.33(46,444,129_51,197,766) ×1 Dn	4.7 Mb	42	Fetal BPD, HC and FL were less than the number of weeks of menopause, bilateral polycystic dysplasia of kidney; bladder not shown; left ventricular strong echo; oligohydramnios	TOP
2	14+	46,XX	arr[hg19]22q13.2q13.31(43,560,872–44,259,781) ×3 VOUS Dn 22q13.31q13.33(44,261,580–51,197,766) ×1 P Dn	699 Kb 6.9 Mb	3 54	NA	Missed abortion
3	23+	46,XY	arr[hg19] 22q13.31q13.33(46,748,488–51,197,766) ×1 Dn	4.4 Mb	35	AMA; polycystic dysplasia of the left fetal kidney. single umbilical artery; cardiac systolic function is briefly reduced	TOP
4	14	46,XY	arr[hg19]8q13.3q24.3(70,949,908–146,295,771) ×2–3(70%) Dn 22q13.31q13.33(48,324,664–51,197,766) ×1–2 (70%) Dn	75.3 Mb 2.3 Mb	487 46	NA	Missed abortion
5	22+	46,XX	arr[hg19] 22q13.33(49,683,904–51,197,766) ×1 Dn	1.5 Mb	29	FGR, intestinal duct echo enhancement	TOP
6	22	46,XX,del(22)(q13.2)	arr[hg19]22q13.2q13.33(43,784,093–51,197,766) ×1 Dn	7.4 Mb	55	AMA, the transverse diameter of the fetal cerebellum was slightly smaller, and the gallbladder was not shown	TOP
7	24+	46,XX	arr[hg19]22q13.2q13.33(42,640,606–51,197,766) ×1 Dn	8.5 Mb	72	FGR, fetal left hydronephrosis, double renal parenchyma echo slightly enhanced, double renal accessory renal artery, local intestinal duct echo slightly enhanced	TOP
8	23	46,XY,del(22) (q13.2)	arr[hg19] 22q13.2q13.33(43,117,811–51,197,766) ×1 Dn	8.0 Mb	65	Fetal VSD, tricuspid valve mild regurgitation, persistent left superior vena cava, bilateral polycystic kidney, left renal accessory renal artery	TOP
9	25+	46,XY	arr[hg19]22q13.33(50,788,194_51,197,766) ×1 P Dn 7q35(144,075,390_145,308,635) ×3 VOUS mat	410 Kb 1.2 Mb	14 3	Fetal VSD, persistent left superior vena cava, tricuspid valve mild regurgitation; bilateral ventriculomegaly, abnormal spinal development (the thoracic vertebrae and ribs are disordered and curved to the left)	TOP
10	13+	46,XX	arr[hg19]22q13.2q13.33(42,765,874_51,168,157) ×1 P Dn	8.4 Mb	76	NA	Missed abortion
11	11+	46,XX	arr[hg19]22q13.2q13.31(41,932,084_44,554,923) ×4, VOUS Dn 22q13.31q13.33(44,555,028_51,197,766) ×1 P Dn	2.6 Mb 6.6 Mb	44 55	NA	Missed abortion

Abbreviations: AMA, advanced maternal age; ASD, autism spectrum disorder; BPD, biparietal diameter; Dn, de novo; FGR, fetal growth restriction; HC, head circumference; FL, femur length; LV, left ventricle; Mat, maternally; NA, not available; OMIM, Online Mendelian Inheritance in Man; P, pathogenic; PLSVC, persistent left superior vena cava; SNP, single nucleotide polymorphism; VOUS, variant of uncertain significance; VSD, ventricular septal defec; TOP, Termination of pregnancy.

**Table 3:** Details of the Phelan-McDermid syndrome detected in postnatal 4 cases.

Postnatal Case	Age at Diagnosis (Years)	Abnormal Phenotype	Karyotype	CNV-Seq Results (CNV Classification, Size, Inheritance)	SNP Array Results (CNV Classification, Size, Inheritance)	CNV Location	WES Results (ACMG Classification, Variant Type, Size, Inheritance)	SNV Location
1	10	GDD, severe language DD, brain MRI examination showed abnormal white matter and delayed myelination	46,XY	NA	arr[hg19]22q13.33 (51,128,325_51,197,766) ×1 P, 69.4 Kb, Dn	Intragenic deletion Exon 12–25	NA	NA
2	9	GDD and language DD, craniofacial deformities (long face shape, wide eye distance, low nose bridge, pointed chin)	46,XY	NA	arr[hg19]22q13.33(50,040,369_51,197,766) ×1 P, 1.1 Mb, Dn	NA	NA	NA
3	3	Moderate ID	46,XX	NA	arr[hg19](1–22)x2,(XY) ×1	NA	[hg19]*SHANK3* (NM_001372044.1)c.3513_3514del (p.L1172H)fs*198 LP(PVS1+PM2) Frameshift NA	Exon 21
4	2.5	Gross motor retardation, ASD, moderate ID, myasthenia, muscular dystrophy	46,XY	seq[hg19]dup(15)(q13.3q13.3) chr15:g.32,020,000_32,460,000dup VOUS, 0.44 Mb Dn	arr[hg19]22q13.33 (51,128,325_51,197,766) ×1 69.4 Kb P Dn	Intragenic deletion Exon 12–25	① [hg38]22q13.33 (50,684,585–50,799,128) ×1 het P, 110 Kb, Dn ② *VMA21* (NM 001363810) c.214C>T(p.R72C missense variant	The intragenic deletion of 3′ end and exons 11 to 25 in *SHANK3* (NM_001372044.2) Exon 1

Abbreviations: ASD, autism spectrum disorder; CNV, copy number variation; DD, developmental delay; Dn, de novo; GDD, global developmental delay; ID, intellectual disability; LP, likely pathogenic; P, pathogenic; SNP, single nucleotide polymorphism; SNV, single nucleotide variant; VOUS, variant of uncertain significance; WES, whole exome sequencing.

## Data Availability

The datasets used and analyzed during the current study are included in Table 1, Table 2, Table 3 and Tables S1–S3 and Figure 1, Figures S1 and S2. Detailed clinical findings on patients will be available by contacting the corresponding authors.

## Supplementary Materials

The following supporting information can be downloaded at https://www.mdpi.com/article/10.3390/jcm15187130/s1: Figure S1: Predicted 3D structures of wild-type and truncated SHANK3; Figure S2: Heatmap of phenotype proportions by gene and clinical system; Table S1: Clinical phenotypic classification of prenatal and postnatal cases based on 10 major system involvement in Phelan-McDermid syndrome; Table S2: Detailed counts of phenotype-positive cases among gene carriers across clinical systems; Table S3: Review of clinical features and diagnostic method in prenatal fetuses diagnosed with PMS during the last 24 years.
