# Supplementary material for: Prenatal and Postnatal Identification of Phelan–McDermid Syndrome in a Tertiary Referral Center: A 15-Case Series and Literature Review
Source: J Clin Med. 2026 Sep 14;15(18):7130. doi: 10.3390/jcm15187130 (PMC13607610; doi:10.3390/jcm15187130)
Supplement: Supplementary file 1 [file jcm-15-07130-s001.zip › jcm-4436180-supplementary.pdf]

Figure S1. Predicted 3D structures of wild-type and truncated SHANK3.

(A) Wild-type SHANK3 (full-length, 1748 aa). Different secondary structural elements are represented in distinct colours (α-helices in red, β-strands in yellow, loops in green). (B) Mutant SHANK3 with c.3513_3514delCC (p.L1172Hfs*198). The frameshift introduces a premature stop at position 1370, resulting in a truncated protein. The red arrow indicates the region where multiple α-helices and β-strands present in the wild-type protein are completely lost in the mutant. Structures were modelled with SwissModel and visualized with PDBviewer.

(**A**)

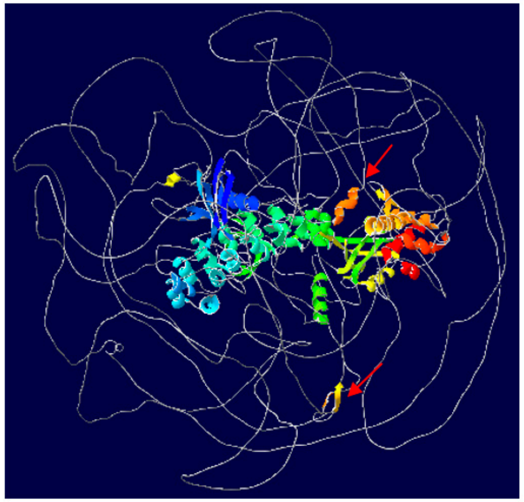

(**B**)

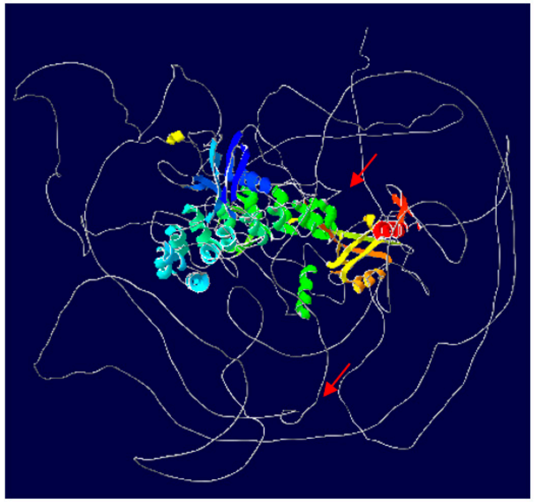

Figure S2. Heatmap of phenotype proportions by gene and clinical system.

Each cell shows the fraction of gene carriers who also have a phenotype in that system. Colour scale: white (0) to dark red (1). Systems on top: CNS, MSK (musculoskeletal), CVS, GUS, GIS, EMS, SO, DF, BG, Others. Genes (y-axis) are in alphabetical order. The colour bar on the right indicates the proportion.

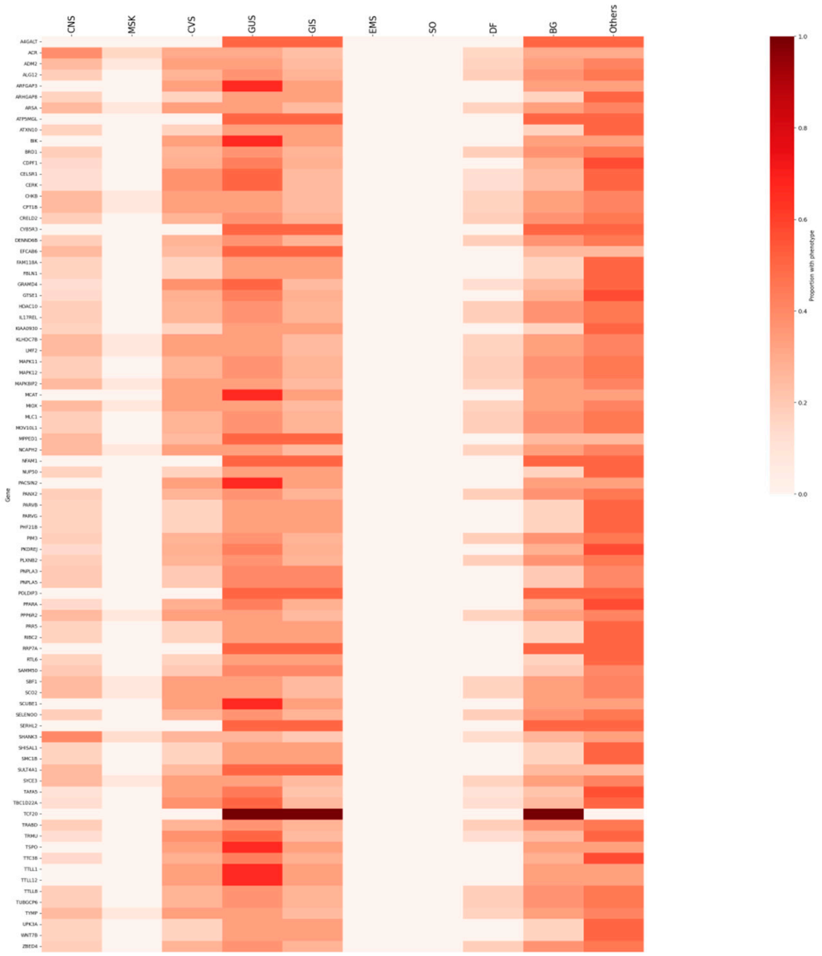

Table S1. Clinical phenotypic classification of prenatal and postnatal cases based on 10 major system involvement in Phelan-McDermid syndrome

Case ID	CNS	MSK	CVS	GUS	GIS	EMS	SO	DF	BG	Others
**Prenatal cases**										
1	NA	NA	Left ventricular hyperechogenic focus	Bilateral polycystic dysplastic kidneys; bladder not visualized	NA	NA	NA	NA	BPD, HC, FL< gestational age	Oligohydramnios
2	NA	NA	NA	NA	NA	NA	NA	NA	NA	Missed abortion
3	NA	NA	Transiently decreased cardiac systolic function	Left fetal polycystic dysplastic kidney	NA	NA	NA	Single umbilical artery	NA	NA
4	NA	NA	NA	NA	NA	NA	NA	NA	NA	Missed abortion
5	NA	NA	NA	NA	Fetal intestinal echogenicity	NA	NA	NA	FGR	NA
6	Fetal cerebellar transverse diameter mildly reduced	NA	NA	NA	Gallbladder not visualized	NA	NA	NA	NA	NA
7	NA	NA	NA	Left hydronephrosis; bilateral hyperechogenic renal parenchyma; bilateral accessory renal arteries	Focal intestinal hyperechogenicity	NA	NA	NA	FGR	NA
8	NA	NA	VSD; mild tricuspid regurgitation; PLSVC	Bilateral polycystic kidneys; left accessory renal artery	NA	NA	NA	NA	NA	NA
9	Bilateral ventriculomegaly	Abnormal spinal developme nt	VSD; PLSVC; mild tricuspid regurgitation	NA	NA	NA	NA	NA	NA	NA
10	NA	NA	NA	NA	NA	NA	NA	NA	NA	Missed abortion
11	NA	NA	NA	NA	NA	NA	NA	NA	NA	Missed abortion

**Postnatal cases**										
1	Global developmental delay; abnormal white matter myelination	NA	NA	NA	NA	NA	NA	NA	NA	NA
2	Intellectual disability; speech delay	NA	NA	NA	NA	NA	NA	Long face; hypertelorism; flat nasal bridge; pointed chin	Physical developmental delay	NA
3	Moderate intellectual disability	NA	NA	NA	NA	NA	NA	NA	NA	NA
4	Gross motor delay; ASD; mild intellectual disability	Myasthenia; muscular dystrophy	NA	NA	NA	NA	NA	NA	NA	NA

Abbreviations: CNS, central nervous system; MSK,Musculoskeletal; CVS, cardiovascular system; GUS, genitourinary system; GIS, gastrointestinal system; EMS, endocrine-metabolic system; SO, sensory organs; DF, dysmorphic features; BG, body growth; VSD, ventricular septal defect; PLSVC, persistent left superior vena cava; FGR, fetal growth restriction; ASD, autism spectrum disorder; NA, no abnormality

Table S2. Detailed counts of phenotype-positive cases among gene carriers across clinical systems.

Gene	CNS	MSK	CVS	GUS	GIS	EMS	SO	DF	BG	Others
A4GALT	0/2	0/2	0/2	1/2	1/2	0/2	0/2	0/2	1/2	1/2
ACR	5/13	2/13	4/13	4/13	3/13	0/13	0/13	2/13	4/13	4/13
ADM2	3/12	1/12	4/12	4/12	3/12	0/12	0/12	2/12	4/12	5/12
ALG12	2/11	0/11	3/11	4/11	3/11	0/11	0/11	2/11	4/11	5/11
ARFGAP3	0/3	0/3	1/3	2/3	1/3	0/3	0/3	0/3	1/3	1/3
ARHGAP8	1/6	0/6	1/6	2/6	2/6	0/6	0/6	0/6	1/6	3/6
ARSA	3/12	1/12	4/12	4/12	3/12	0/12	0/12	2/12	4/12	5/12
ATP5MGL	0/2	0/2	0/2	1/2	1/2	0/2	0/2	0/2	1/2	1/2
ATXN10	1/6	0/6	1/6	2/6	2/6	0/6	0/6	0/6	1/6	3/6
BIK	0/3	0/3	1/3	2/3	1/3	0/3	0/3	0/3	1/3	1/3
BRD1	2/11	0/11	3/11	4/11	3/11	0/11	0/11	2/11	4/11	5/11
CDPF1	1/7	0/7	2/7	3/7	2/7	0/7	0/7	0/7	2/7	4/7
CELSR1	1/8	0/8	3/8	4/8	2/8	0/8	0/8	1/8	2/8	4/8
CERK	1/8	0/8	3/8	4/8	2/8	0/8	0/8	1/8	2/8	4/8
CHKB	3/12	1/12	4/12	4/12	3/12	0/12	0/12	2/12	4/12	5/12
CPT1B	3/12	1/12	4/12	4/12	3/12	0/12	0/12	2/12	4/12	5/12
CRELD2	2/11	0/11	3/11	4/11	3/11	0/11	0/11	2/11	4/11	5/11
CYB5R3	0/2	0/2	0/2	1/2	1/2	0/2	0/2	0/2	1/2	1/2
DENND6B	2/11	0/11	3/11	4/11	3/11	0/11	0/11	2/11	4/11	5/11
EFCAB6	1/4	0/4	1/4	2/4	2/4	0/4	0/4	0/4	1/4	1/4
FAM118A	1/6	0/6	1/6	2/6	2/6	0/6	0/6	0/6	1/6	3/6
FBLN1	1/6	0/6	1/6	2/6	2/6	0/6	0/6	0/6	1/6	3/6
GRAMD4	1/8	0/8	3/8	4/8	2/8	0/8	0/8	1/8	2/8	4/8
GTSE1	1/7	0/7	2/7	3/7	2/7	0/7	0/7	0/7	2/7	4/7

HDAC10	2/11	0/11	3/11	4/11	3/11	0/11	0/11	2/11	4/11	5/11
IL17REL	2/11	0/11	3/11	4/11	3/11	0/11	0/11	2/11	4/11	5/11
KIAA0930	1/6	0/6	1/6	2/6	2/6	0/6	0/6	0/6	1/6	3/6
KLHDC7B	3/12	1/12	4/12	4/12	3/12	0/12	0/12	2/12	4/12	5/12
LMF2	3/12	1/12	4/12	4/12	3/12	0/12	0/12	2/12	4/12	5/12
MAPK11	2/11	0/11	3/11	4/11	3/11	0/11	0/11	2/11	4/11	5/11
MAPK12	2/11	0/11	3/11	4/11	3/11	0/11	0/11	2/11	4/11	5/11
MAPK8IP2	3/12	1/12	4/12	4/12	3/12	0/12	0/12	2/12	4/12	5/12
MCAT	0/3	0/3	1/3	2/3	1/3	0/3	0/3	0/3	1/3	1/3
MIOX	3/12	1/12	4/12	4/12	3/12	0/12	0/12	2/12	4/12	5/12
MLC1	2/11	0/11	3/11	4/11	3/11	0/11	0/11	2/11	4/11	5/11
MOV10L1	2/11	0/11	3/11	4/11	3/11	0/11	0/11	2/11	4/11	5/11
MPPED1	1/4	0/4	1/4	2/4	2/4	0/4	0/4	0/4	1/4	1/4
NCAPH2	3/12	1/12	4/12	4/12	3/12	0/12	0/12	2/12	4/12	5/12
NFAM1	0/2	0/2	0/2	1/2	1/2	0/2	0/2	0/2	1/2	1/2
NUP50	1/6	0/6	1/6	2/6	2/6	0/6	0/6	0/6	1/6	3/6
PACSIN2	0/3	0/3	1/3	2/3	1/3	0/3	0/3	0/3	1/3	1/3
PANX2	2/11	0/11	3/11	4/11	3/11	0/11	0/11	2/11	4/11	5/11
PARVB	1/6	0/6	1/6	2/6	2/6	0/6	0/6	0/6	1/6	3/6
PARVG	1/6	0/6	1/6	2/6	2/6	0/6	0/6	0/6	1/6	3/6
PHF21B	1/6	0/6	1/6	2/6	2/6	0/6	0/6	0/6	1/6	3/6
PIM3	2/11	0/11	3/11	4/11	3/11	0/11	0/11	2/11	4/11	5/11
PKDREJ	1/7	0/7	2/7	3/7	2/7	0/7	0/7	0/7	2/7	4/7
PLXNB2	2/11	0/11	3/11	4/11	3/11	0/11	0/11	2/11	4/11	5/11
PNPLA3	1/5	0/5	1/5	2/5	2/5	0/5	0/5	0/5	1/5	2/5
PNPLA5	1/5	0/5	1/5	2/5	2/5	0/5	0/5	0/5	1/5	2/5

POLDIP3	0/2	0/2	0/2	1/2	1/2	0/2	0/2	0/2	1/2	1/2
PPARA	1/7	0/7	2/7	3/7	2/7	0/7	0/7	0/7	2/7	4/7
PPP6R2	3/12	1/12	4/12	4/12	3/12	0/12	0/12	2/12	4/12	5/12
PRR5	1/6	0/6	1/6	2/6	2/6	0/6	0/6	0/6	1/6	3/6
RIBC2	1/6	0/6	1/6	2/6	2/6	0/6	0/6	0/6	1/6	3/6
RRP7A	0/2	0/2	0/2	1/2	1/2	0/2	0/2	0/2	1/2	1/2
RTL6	1/6	0/6	1/6	2/6	2/6	0/6	0/6	0/6	1/6	3/6
SAMM50	1/5	0/5	1/5	2/5	2/5	0/5	0/5	0/5	1/5	2/5
SBF1	3/12	1/12	4/12	4/12	3/12	0/12	0/12	2/12	4/12	5/12
SCO2	3/12	1/12	4/12	4/12	3/12	0/12	0/12	2/12	4/12	5/12
SCUBE1	0/3	0/3	1/3	2/3	1/3	0/3	0/3	0/3	1/3	1/3
SELENOO	2/11	0/11	3/11	4/11	3/11	0/11	0/11	2/11	4/11	5/11
SERHL2	0/2	0/2	0/2	1/2	1/2	0/2	0/2	0/2	1/2	1/2
SHANK3	6/15	2/15	4/15	4/15	3/15	0/15	0/15	2/15	4/15	5/15
SHISAL1	1/6	0/6	1/6	2/6	2/6	0/6	0/6	0/6	1/6	3/6
SMC1B	1/6	0/6	1/6	2/6	2/6	0/6	0/6	0/6	1/6	3/6
SULT4A1	1/4	0/4	1/4	2/4	2/4	0/4	0/4	0/4	1/4	1/4
SYCE3	3/12	1/12	4/12	4/12	3/12	0/12	0/12	2/12	4/12	5/12
TAFA5	1/9	0/9	3/9	4/9	2/9	0/9	0/9	1/9	2/9	5/9
TBC1D22A	1/8	0/8	3/8	4/8	2/8	0/8	0/8	1/8	2/8	4/8
TCF20	0/1	0/1	0/1	1/1	1/1	0/1	0/1	0/1	1/1	0/1
TRABD	2/11	0/11	3/11	4/11	3/11	0/11	0/11	2/11	4/11	5/11
TRMU	1/8	0/8	3/8	4/8	2/8	0/8	0/8	1/8	2/8	4/8
TSPO	0/3	0/3	1/3	2/3	1/3	0/3	0/3	0/3	1/3	1/3
TTC38	1/7	0/7	2/7	3/7	2/7	0/7	0/7	0/7	2/7	4/7
TTLL1	0/3	0/3	1/3	2/3	1/3	0/3	0/3	0/3	1/3	1/3

TTLL12	0/3	0/3	1/3	2/3	1/3	0/3	0/3	0/3	1/3	1/3
TTLL8	2/11	0/11	3/11	4/11	3/11	0/11	0/11	2/11	4/11	5/11
TUBGCP6	2/11	0/11	3/11	4/11	3/11	0/11	0/11	2/11	4/11	5/11
TYMP	3/12	1/12	4/12	4/12	3/12	0/12	0/12	2/12	4/12	5/12
UPK3A	1/6	0/6	1/6	2/6	2/6	0/6	0/6	0/6	1/6	3/6
WNT7B	1/6	0/6	1/6	2/6	2/6	0/6	0/6	0/6	1/6	3/6
ZBED4	2/11	0/11	3/11	4/11	3/11	0/11	0/11	2/11	4/11	5/11

Note: The cell shows the number of cases with the phenotype (numerator) over the total number of cases carrying the gene (denominator). Systems: central nervous system (CNS), musculoskeletal (MSK), cardiovascular (CVS), genitourinary (GUS), gastrointestinal (GIS), endocrine/metabolic (EMS), sensory organs (SO), dysmorphic features (DF), blood/immune (BG), and Others.

Table S3. Review of clinical features and diagnostic method in prenatal fetuses diagnosed with PMS during the last 24 years.

Study	Weeks at PD	Genetic test technology	Indication of PD	Karyotype results	CMA results	Inheritance	Obstetric outcome
Chen et al.(2025)[A]	30	Karyotype, aCGH,	Fetal severe right hydronephrosis and hydroureter	46,XX,r(22)(p11.2q13.31)	arr[hg19] 22q13.31q13.33 (45,736,280e51,178,264)x1	Dn	TOP
Hao et al.(2022)[B]	The second trimester	Karyotype and CMA	Increased risk of Down syndrome by maternal serum screening, abnormal finding in the noninvasive prenatal testing	46,XN	arr[hg19]22q13.33 (49628163_51197766)x1	Dn	TOP
	The second trimester	Karyotype and CMA	Fetal growth restriction, single umbilical artery, complex congenital heart disease, jejunal atresia, cystic dysplasia of the left kidney, obstructive reflux renal injury, and persistent left superior vena cava	46,XN,del(22) (q13.2)	arr[hg19] 22q13.2q13.33 (44164976_51197766)x1	Dn	TOP
	The second trimester	Karyotype and CMA	Increased risk of Down syndrome by maternal serum screening, advanced maternal age	46,XN	arr[hg19]22q13.33 (51128325_51197766)x1	Dn	TOP
	The second trimester	Karyotype and CMA	Nuchal translucence	46,XN	arr[hg19]22q13.33 (51128324_51197766)x1	Dn	TOP
	The second trimester	Karyotype and CMA	Parental carriers, nuchal translucence	46,XN,der (22) t(15;22)(p11.2;q13.31)pat	arr[hg19] 22q13.31q13.33 (47220048_51197766)x1	pat	TOP
	The second trimester	Karyotype and CMA	Fetal growth restriction, single umbilical artery, complex congenital heart disease, jejunal atresia, cystic dysplasia of the left	46,XN,del (22) (q13)	arr[hg19] 22q13.31q13.33 (46102178_51197766)x1	Dn	TOP

			kidney, obstructive reflux renal injury, and persistent left superior vena cava				
	The second trimester	Karyotype and CMA	Multiple structural abnormalities(morphological changes of skull, local premature closure of coronal suture, short long bones, early ossification of distal femur epiphyseal cartilage, abnormal rib shape, enlarged cardiothoracic ratio, polycystic dysplasia of the right kidney, and the dermal medulla of the left kidney is unclear)	46,XN,del (22) (q13)	arr[hg19] 22q13.2q13.33 (42135216_51197766)x1	Dn	TOP
Shen et al.(2020)[C]	The second trimester	Karyotype, SNP array, and FISH	Fetal ventricular septal defect, choroid cyst, persistent left superior vena cava, mild regurgitation of tricuspid valve, right ventricular choroid cyst	46,XX	arr [hg19] 22q13.31g13.33(46009655.51197766)x1,21q21.1g21.2(20088087-24600640)x1	Dn	TOP
Ge et al. (2020) [D]	23	Karyotype, SNP array, and FISH	NIPT shows a 5.37 Mb microduplication of chromosome 22; fetal choroid plexus cyst in the right ventricle, bilateral renal pelvis separation, and single umbilical artery	46,X?	arr[hg19] 22q13.2q13.33 (44088529-51197766)x1	Dn	TOP
	22	Karyotype, SNP array, and FISH	Fetal increased risk of Down"s syndrome by maternal serum screening	46,X?[48]/46,X?,+ marl,-22[9]/46.X?,+mar2,-22[3]	arr[hg19]22q13.31q13.33(44,620,384-51,197,766)x1, 22q13.32q13.33(48,916,416-51,197,766)x1-2, 22q11.1q13.2(16,888,899-42,985.310)x2-3	Dn	CTP
Luo et al. (2019) [E]	25	SNP array, MLPA, and FISH	NIPT shows microduplication of	46,X?	arr[hg19] 22q13.31q13.33 (47167879-	Dn	TOP

			chromosome 11*		51197838)x1		
Phelan MC, et al(2001)[F]	17	Karyotype and FISH	Increased risk of DS	mosaicism 46,XX,del(22)(q13)	NA	Dn	TOP
Riegel M, et al (2000) [G]	21	Karyotype and FISH	Fetal cystic 'tumour' in the fetal neck and the upper thoracic aperture	mosaicism 46,XX,del(22)(q13)	NA	Dn	TOP

Abbreviations: PD, prenatal diagnosis;AMA, advanced maternal age; NIPT, noninvasive prenatal testing; FGR, fetal growth restriction; Dn, de novo; DS, down syndrome

aCGH, array comparative genomic hybridization (aCGH); FISH, fluorescence in situ hybridization; TOP,termination of pregnancy; CTP, continue the pregnancy; CMA, chromosomal microarray analysis; TB, term birth; SNP, single nucleotide polymorphism; FISH, fluorescence in situ hybridizatio; MLPA, Multiplex ligation-dependent probe amplification

*There were no obvious deformities in the face and limbs of the induced male fetus. The pregnant couple refused the pathological examination.thus, it is unknown whether there were any abnormalities in the internal organ structure.

[A] Chen, Chih-Ping et al. “Prenatal diagnosis of a 5.44-Mb de novo 22q13.31q13.33 deletion encompassing SHANK3 associated with mosaicism for r(22)(p11.2q11.31) and monosomy 22 in a fetus with severe right hydronephrosis and hydroureter on ultrasound and determination of a maternal origin of the deletion and r(22) by quantitative fluorescent polymerase chain reaction.” Taiwanese journal of obstetrics & gynecology vol. 64,2 (2025): 334-338. doi:10.1016/j.tjog.2024.12.011.

[B] Hao, Ying et al. “Prenatal and postnatal diagnosis of Phelan-McDermid syndrome: A report of 21 cases from a medical center and review of the literature.” Frontiers in genetics vol. 13 961196. 31 Aug. 2022, doi:10.3389/fgene.2022.961196.

[C] Shen, H. X., Li, S. P., and Jin, Y. X. (2020). [Prenatal diagnosis of a fetus with Phelan-McDermid syndrome and 21q21 microdeletion by multiple genetic techniques]. Zhonghua Yi Xue Yi Chuan Xue Za Zhi 37, 1387–1390. doi:10.3760/cma.j.cn511374-20200303-00123.

[D] Ge, Y. S., Zhang, J., Cai, M. J., Chen, X. L., and Zhou, Y. L. (2020). Prenatal genetic analysis of three fetuses with abnormalities of chromosome 22. Zhonghua Yi Xue Yi Chuan Xue Za Zhi 37, 405–409. doi:10.3760/cma.j.issn.1003-9406.2020.04.010.

[E] Luo, Y. Q., Qian, Y. Q., Wang, L. Y., Yang, Y. M., Sun, Y. X., Jin, F., et al. (2019). Prenatal diagnosis of a fetus with Phelan-McDermid syndrome. Zhonghua Yi Xue Yi Chuan Xue Za Zhi 36, 841–843. doi:10.3760/cma.j.issn.1003-9406.2019.08.022.

[F] Phelan MC, Brown EF, Rogers RC. Prenatal diagnosis of mosaicism for deletion 22q13.3. Prenat Diagn. 2001;21(12):1100. doi:10.1002/pd.195.

[G] Riegel M, Baumer A, Wisser J, Acherman J, Schinzel A. Prenatal diagnosis of mosaicism for a del(22)(q13). Prenat Diagn. 2000;20(1):76-79. doi:10.1002/(sici)1097-0223(200001)20:1<76::aid-pd752>3.0.co;2-m.
